# Supplementary material for: Aberrant APOBEC3B Expression in Breast Cancer Is Linked to Proliferation and Cell Cycle Phase
Source: Cells. 2023 Apr 18;12(8):1185. doi: 10.3390/cells12081185 (PMC10136826; doi:10.3390/cells12081185)
Supplement: Supplementary file 1 [file cells-12-01185-s001.zip › Supplementary Figures.pdf]

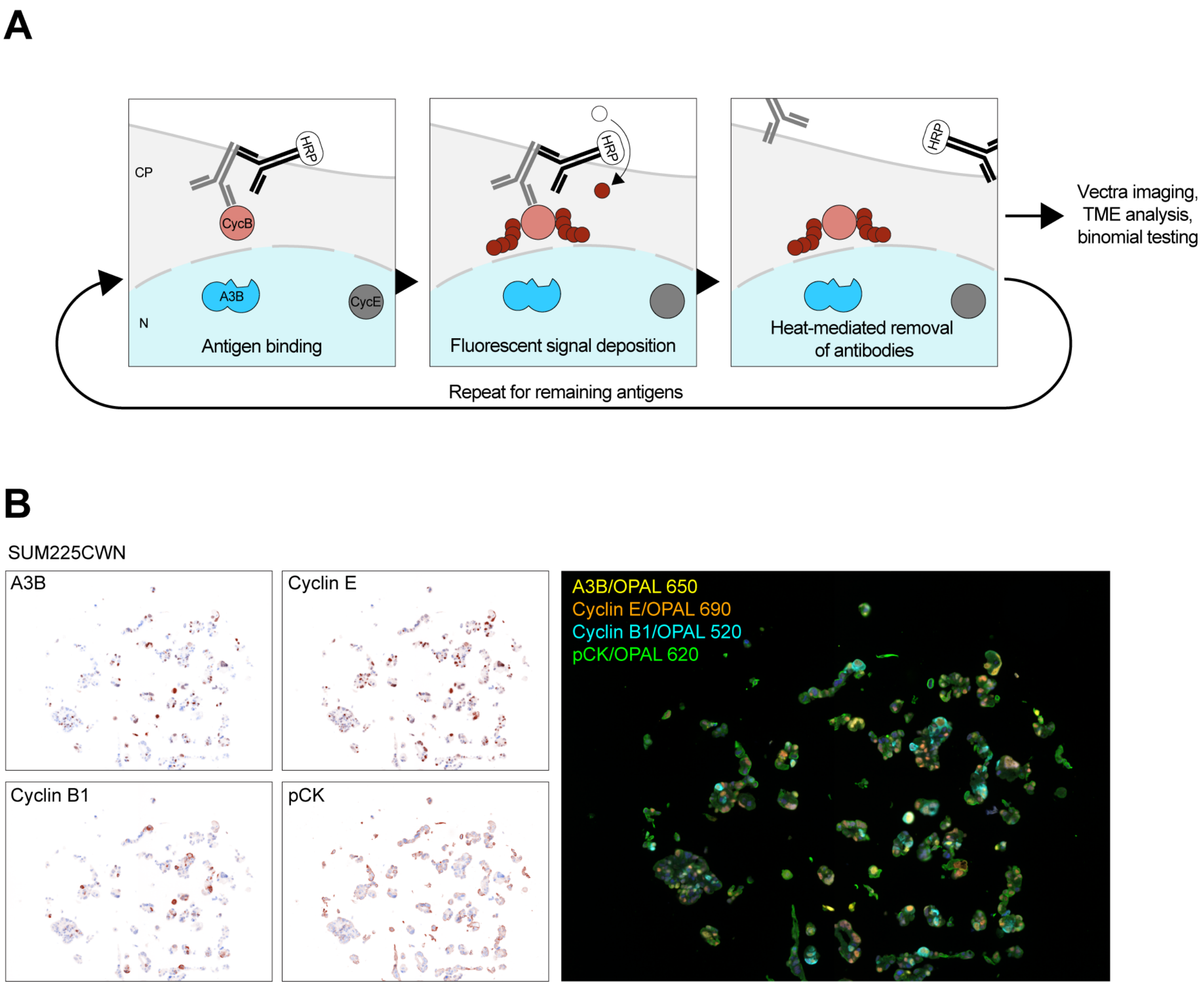

Supplementary Figure S1. Additional information regarding multiplex IHC.

A Schematic overview of the workflow used during multiplex IHC, which involves the sequential binding of antigens by monoclonal primary antibodies, the development and deposition of fluorescent signal through HRP conjugated secondary antibodies, and the heat-mediated removal of both primary and secondary antibodies. After completion, slides were analyzed (see Methods) and data was subjected to binomial testing using an in-house Python script.

B Representative microscope field-of-view during image acquisition using multiplex IHC for A3B, Cyclin E2, Cyclin B1, and pan CK. An example of the breast cancer cell line SUM225CWN is shown. Note that while fluorescent markers are used for signal deposition, images used for display of individual markers are pseudo-colored to emulate chromogenic staining patterns. Pan cytokeratin was used to determine cell boundaries after which pair-wise comparisons were made between A3B and individual Cyclins. The image on the right represents a composite view of all four channels pseudo-colored in yellow (A3B), orange (Cyclin E), cyan (Cyclin B1), and green (pan CK)

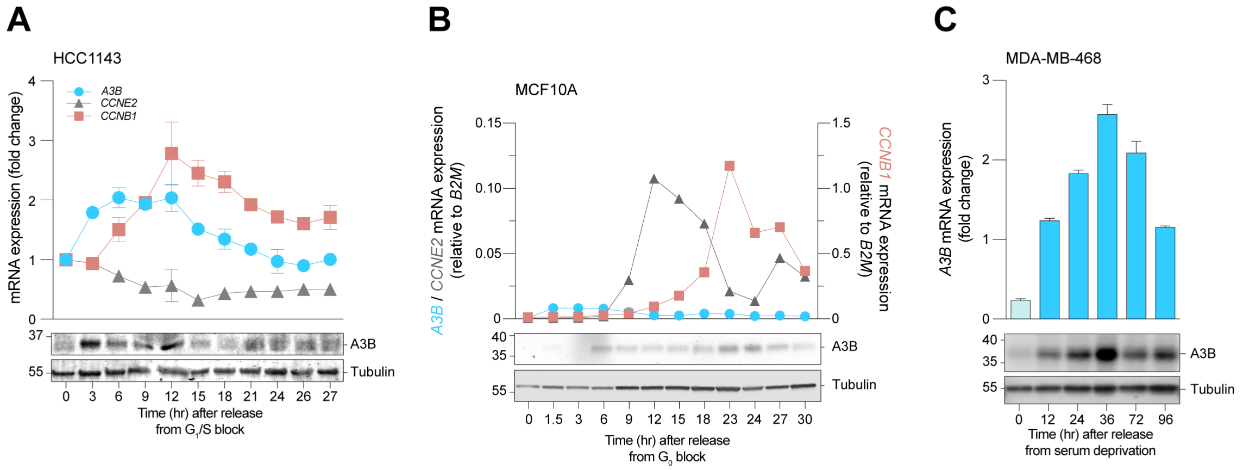

Supplementary Figure S2. Detailed time-courses and MDA-MB-468 serum deprivation A-B RT-qPCR for *A3B*, *CCNE2*, and *CCNB1* (top) and A3B immunoblot (bottom) in HCC1143 and MCF10A, taken at the indicated times following a double thymidine block or serum starvation, respectively. These data represent the full extent of the HCC1143 and MCF10A samples used in the experiment described in Figure 2B-C and additionally show A3B protein expression.

C RT-qPCR (top) and immunoblot (bottom) for A3B expression performed on MDA-MD-468 cells at various hours following serum deprivation.

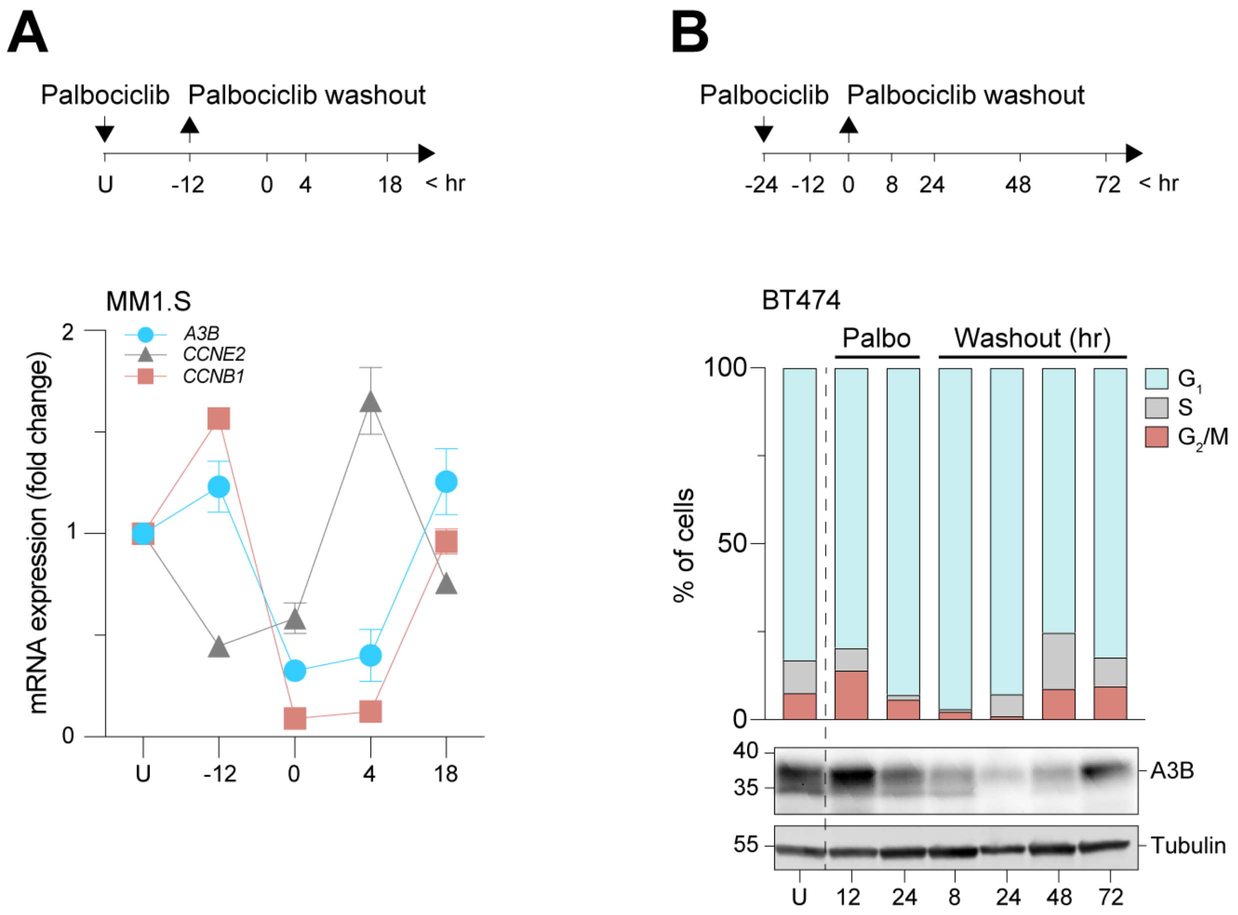

Supplementary Figure S3. Additional cell cycle synchronization courses using palbociclib

A Reanalyzed *A3B*, *CCNE2*, and *CCNB1* expression data in the multiple myeloma cell line MM1.S as sampled before, during, and after synchronization using palbociclib. Publicly available data (GSE35728) was used.

B Additional time course of BT474 synchronized using palbociclib followed by an extended outgrowth period (see top panel). Cell cycle progression, as analyzed by PI stain, and an A3B immunoblot are shown.

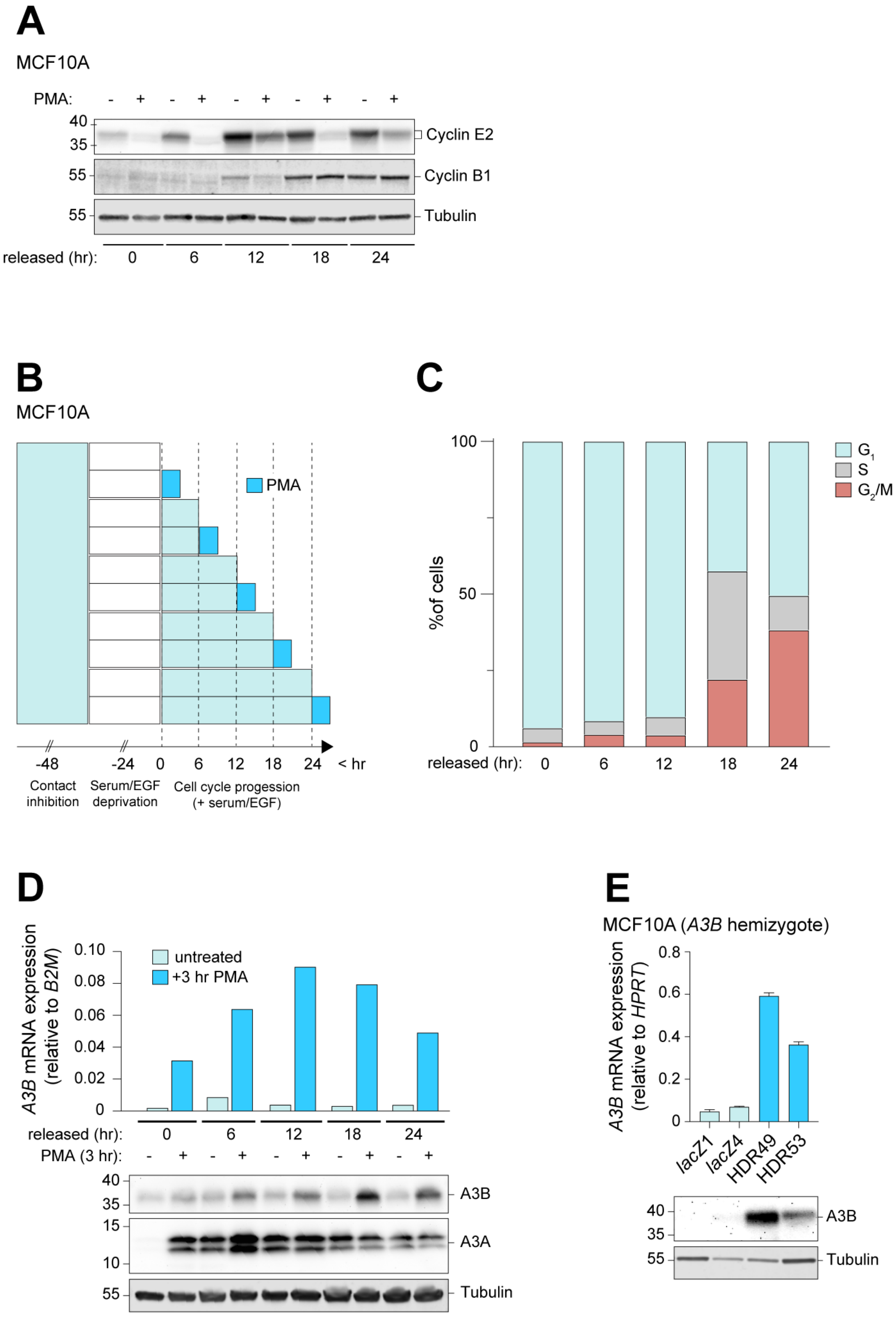
A
MCF10A
PMA:
- + - + - + - + - +
40
35
Cyclin E2
55
Cyclin B1
55
Tubulin
released (hr): 0 6 12 18 24
B
MCF10A
PMA
-48
-24
0
6
12
18
24
< hr
Contact inhibition
Serum/EGF deprivation
Cell cycle progession (+ serum/EGF)
C
100
50
0
%of cells
G1
S
G2/M
released (hr): 0 6 12 18 24
D
A3B mRNA expression (relative to B2M)
0.10
0.08
0.06
0.04
0.02
0
untreated
+3 hr PMA
released (hr): 0 6 12 18 24
PMA (3 hr): - + - + - + - + - +
40
35
A3B
15
10
A3A
55
Tubulin
E
MCF10A (A3B hemizygote)
A3B mRNA expression (relative to HPRT)
0.8
0.6
0.4
0.2
0
lacZ1
lacZ4
HDR49
HDR53
40
35
A3B
55
Tubulin

Supplementary Figure S4. Additional cell cycle synchronization courses using palbociclib

A Supporting immunoblots showing the expression of Cyclin E2 and Cyclin B1 before and after PMA treatment throughout the experiments described in Figure 4B-D. Note that Cyclin E2 expression is consistently impacted by PMA treatment.

B-D Additional cell cycle experiment, with minor adaptations as compared to Figure 4B-D. Note that in this experiment, PMA treated cells were compared to control samples taken at the time of PMA addition (B). Cell cycle progression was measured by flowcytometry (C). RT-qPCR and immunoblot assays were performed to analyze A3B expression in response to PMA treatment (D). A3A expression was included as a control for the general inducibility of genes under control of the PKC/ncNF-κB pathway.

E A3B mRNA (top) and protein (bottom) expression of two *lacZ* control (light blue) and HDR clones (blue). The HDR clones in this study possess a deactivating mutation in the repressive E2F element within the *A3B* promoter.

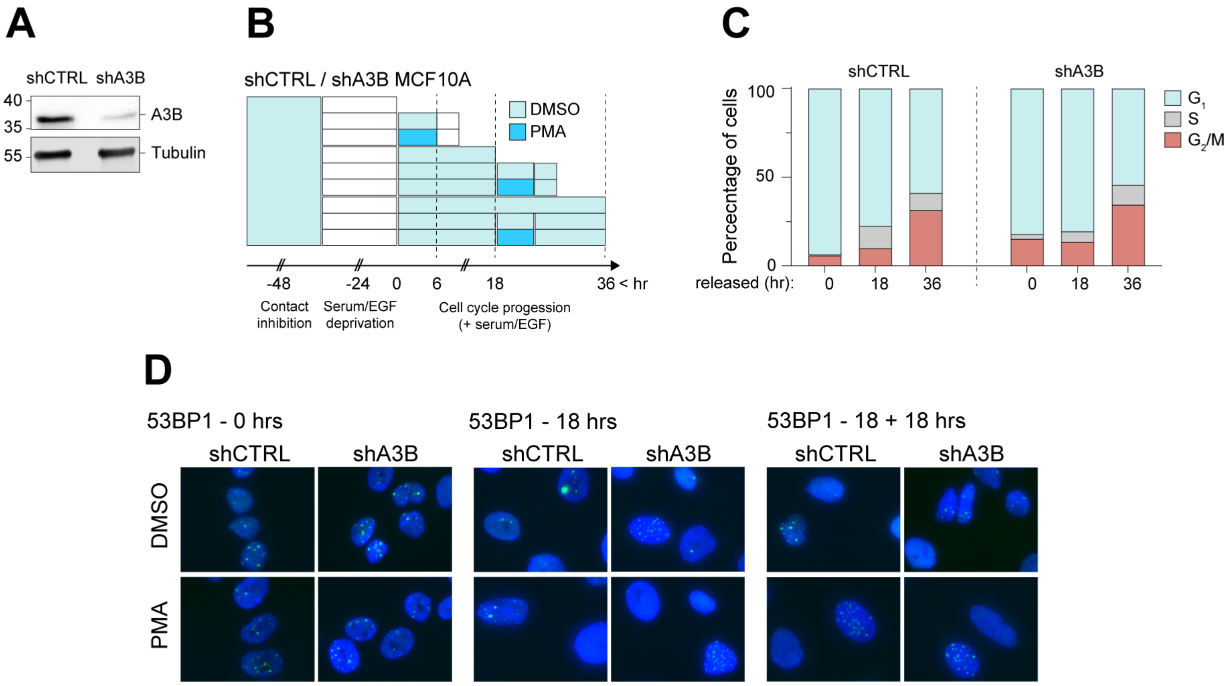

Supplementary Figure S5. PMA induction of A3B and DNA damage response

A Immunoblots showing A3B expression in MCF10A cells after transduction with shCTRL and shA3B lentiviral particles and puromycin selection.

B Treatment regimen used to investigate the activation of the DNA damage response upon PMA treatment in growth-arrested cells, cells released for 18 hrs followed by a 3 hr outgrowth period (early response), and cells released for 18 hours followed by a 12 hr outgrowth period (delayed response).

C Flowcytometry plots showing cell cycle progression of both cell lines upon release in normal growth medium.

D Fluorescent microscopy images showing 53BP1 foci after PMA treatment in growth-arrested cells and cells released for 18 hrs (followed by a 3 or 12 hr outgrowth period).
