# Supplementary material for: Aberrant APOBEC3B Expression in Breast Cancer Is Linked to Proliferation and Cell Cycle Phase
Source: Cells. 2023 Apr 18;12(8):1185. doi: 10.3390/cells12081185 (PMC10136826; doi:10.3390/cells12081185)
Supplement: Supplementary file 1 [file cells-12-01185-s001.zip › Suppl_table.pdf]

Supplementary Table S1: Relevant characteristics of the main cell lines used in this study.

**Cell Line**	**Type**	**A3B level**	**Synchronizable by double thymidine block?**	**Alternative synchronization option**	**A3B inducibility by PMA**
**MCF10A**	Immortalized (normal-like)	Very low	No (results in cell death)	Contact inhibition followed by mitogen deprivation	High during proliferation
**MCF7**	Breast cancer	Low	Yes	Not tested	Limited, during proliferation
**HCC1954**	Breast cancer	Low/intermediate	Yes	Not tested	Not tested
**BT474**	Breast cancer	Intermediate	No (proliferation rate too low)	Palbociclib	Not tested
**HCC1143**	Breast cancer	High	Yes	Not tested	Limited, during proliferation
